# A rat model of mild, reversible bortezomib-induced peripheral neuropathy: behavioral resolution despite persistent glial activation

**DOI:** 10.1016/j.ynpai.2026.100240

**Published:** 2026-10-27

**Authors:** Mona Hashemian, Elisabete Bóf Ramos, Mariam Sobhy Atalla, Nadine Cebulla, Jonas Huhn, Oliver Scherf-Clavel, Grit Hein, K. Martin Kortüm, Heike Rittner, Claudia Sommer

**Affiliations:** a Department of Neurology, University Hospital Würzburg, Würzburg, Germany; b Department of Anesthesiology, University Hospital Würzburg, Würzburg, Germany; c Department of Pharmacy, Ludwigs-Maximilians-University of Munich, Munich, Germany; d Department of Psychiatry, Psychosomatic and Psychotherapy, Translational Social Neuroscience Unit, Würzburg, Germany; e Department of Internal Medicine II, University Hospital Würzburg, Würzburg, Germany

**Keywords:** Bortezomib-induced peripheral neuropathy, Resolution, Satellite glial cells, Neurofilament light, Serum BTZ, Calcitonin gene-related peptide

## Abstract

**Background and aims:**

Bortezomib as a first-line treatment for multiple myeloma leads to bortezomib-induced peripheral neuropathy (BIPN), a dose-limiting side effect. We aimed to characterize the temporal course of BIPN and to investigate molecular and cellular changes associated with its development and resolution.

**Methods:**

Male Wistar rats received intraperitoneal injections of either 5% dimethyl sulfoxide (DMSO, vehicle) or bortezomib (0.2 mg/kg) on days 1, 4, 8, and 11 and hypersensitivity was monitored. Sciatic nerves were assessed for morphological changes and levels of interleukin (IL)-6 and IL-1β, and skin was examined for innervation density. Serum was analysed for neurofilament light (NfL) and BTZ concentrations. Dorsal root ganglia were examined for neuronal and satellite glial cell (SGC) activation.

**Results:**

Bortezomib induced mild mechanical allodynia that peaked at day 12 and resolved by day 25, whereas DMSO caused transient hypersensitivity. No reduction of skin innervation was detected in either group. Serum bortezomib was detectable within 30 min post-injection until day 25. The sciatic nerves showed moderate macrophage presence, but no elevated levels of IL-6 and IL-1β. Dorsal root ganglion calcitonin gene-related peptide immunoreactive neurons increased on day 12 in the bortezomib group. Serum NfL remained unchanged across groups. SGC activation was present in both bortezomib and DMSO-treated rats and persisted at day 25.

**Interpretation:**

This low-dose, reversible BIPN model entailed transient hypersensitivity and neuronal activation without axonal loss. Persistent activation of SGC indicated a glial response despite the absence of detectable structural neuronal damage. DMSO effects should be considered when interpreting preclinical pain models.

## Highlights

• Low-dose bortezomib caused mild but lasting touch sensitivity without major nerve damage which would resolve by day 25.
• DMSO alone triggered short-term pain-like behavior.
• Loss of function in nerve fibers without a reduction in fiber density in the skin in either group.
• Bortezomib increased pain-related CGRP-positive neurons early, however glial activation was more pronounced by day 25 in the absence of allodynia.
• Bortezomib was detectable in blood for up to 25 days, no rise in nerve injury marker NfL or key inflammatory cytokines.

## 1 Introduction

The proteasome inhibitor bortezomib (BTZ) is a key treatment for multiple myeloma, significantly improving patient survival rates (Scott et al., 2016). However, about 30% to 40% of patients (Field-Smith et al., 2006), and even up to 80% upon careful investigation (Cebulla et al., 2023), develop a peripheral neuropathy that is characterized by numbness, paraesthesia, and burning pain. Knowledge on the pathophysiology of BTZ-induced peripheral neuropathy (BIPN) is still incomplete, which is why preclinical models have been created to identify possible treatment targets (Duggett and Flatters, 2017). Prior studies have repeatedly demonstrated that in rodent models, BTZ treatment causes dose-dependent mechanical hypersensitivity (Duggett and Flatters, 2017; De Logu et al., 2017; Carozzi et al., 2013). In mice, decreased mechanical thresholds were observed as early as 6 h following a single dose of BTZ at 1 mg/kg (i.p.) and persisted for up to 11 days (De Logu et al., 2017). In rats, 0.1 or 0.2 mg/kg BTZ (i.v.) administered intermittently on days 0, 3, 7, and 10 caused dose-dependent mechanical hypersensitivity that lasted for 28–52 days and 52–80 days, respectively (Duggett and Flatters, 2017). However, with the same administration schedule, another study reported mild mechanical sensitivity upon a BTZ cumulative dose of 0.8 mg/kg (i.p.), which recovered by day 25 from the beginning of the experiment (Yamamoto et al., 2015). The effects of BTZ on cold and heat pain sensitivity have been less consistent. BTZ treatment has been linked to the development of cold allodynia in some studies (Yamamoto et al., 2015; Atalla et al., 2026), but not in others (Duggett and Flatters, 2017).

Glial cells maintain synaptic function by regulating the growth, maturation, disposal, and maintenance of synapses through a range of contact-mediated and diffusible signals. They maintain extracellular potassium ions (K+), glutamate, and water at equilibrium, thereby supporting neuronal metabolism (Ji et al., 2019). Through the production of inflammatory mediators and close neuron-glia interactions inside sensory ganglia, satellite glial cells (SGC) contribute to neuronal sensitization and neuropathic pain (Schulte et al., 2023). SGC in the DRG and Schwann cells in the sciatic nerve have been shown to be severely impacted by BIPN, while the effect on neurons was minor (Cavaletti et al., 2007). Spinal astrocytes, which serve as homeostasis preservers, are activated following 0.6 mg/kg cumulative BTZ treatment, likely through the reduction of glutamate/aspartate transporter (GLAST) (Robinson and Dougherty, 2015). Astrocytes displayed hypertrophy and increased branching, as indicated by GFAP staining, starting at day 7, peaking at day 30 after BTZ injection (Robinson et al., 2014) with concomitant elevations in protein levels of the inflammatory cytokines tumor necrosis factor alpha (TNF-α) and IL-1β (Stockstill et al., 2018). The degree of mechanical hypersensitivity appears to be closely correlated with the presence, intensity, and resolution of GFAP upregulation (Robinson et al., 2014). In the spinal cord of rats injected with BTZ, astrocyte activation and macrophage infiltration have been linked to concurrent elevation of IL-1ß. In sciatic nerve and DRG a reduction of the anti-inflammatory cytokine IL-10 was found, while TNF-α and IL-6, in addition to IL-1ß mRNA were increased (Moschetti et al., 2019), in parallel to activation of transcription factor 3 (ATF3) (Liu et al., 2016).

Medium or small-diameter sensory neurons of the DRG, especially those involved in nociception, are the primary source of calcitonin gene-related peptide (CGRP), a neuropeptide with 37 amino acids (Priestley et al., 2002; Iyengar et al., 2017). Through its involvement in both central and peripheral sensitization pathways, CGRP leads to a persistence of painful neuropathic symptoms (Iyengar et al., 2017; Quartu et al., 2014). CGRP triggers the expression of CLR/RAMP1 on spinal neurons and Schwann cells in peripheral nerves. This activation has a role in neurogenic inflammation and peripheral sensitization, both of which are essential for the onset and maintenance of neuropathic pain symptoms such as hyperalgesia and mechanical allodynia (Russell et al., 2014; De Logu et al., 2022).

Neurofilament light chain (NfL) is released into the bloodstream after neuronal damage and has been used as a biomarker for axonal injury (Meregalli et al., 2020). NfL has been demonstrated to increase in response to BTZ treatment, and patients with BIPN have higher serum NfL levels, which are correlated with the severity of neuropathic pain (Cebulla et al., 2023).

There are different models for BIPN induction in animals, and the time point of the molecular and behavioral resolution differs based on separate models. Here, we aimed to study the resolution of molecular alterations in addition to behavioral outcomes using a BIPN model previously described (Yamamoto et al., 2015). Having used 5% DMSO as vehicle in our study, following previous protocols, we also report on some unexpected findings related to DMSO.

## 2 Materials and methods

### 2.1 Drugs

BTZ (PS-341) and isoflurane were purchased from Selleck Chemicals (Cologne, Germany) and Dechra (Aulendorf, Germany), respectively. BTZ was dissolved in DMSO 5% (in saline) which was obtained from Sigma-Aldrich (USA). Methanol, formic acid, and acetonitrile were obtained from Sigma-Aldrich. Ultrapure water was generated from a water purification system (MilliQ Ultrapure Water Systems).

### 2.2 Animals

Adult male Wistar rats (eight weeks, 150–200 g) were purchased from Janvier Labs (France) and maintained in a controlled environment (12 h light/dark cycles and ambient temperature of 22 °C) with food and water ad libitum. Based on the 3R protocol, we strived to minimize the number of animals used. The experiments were approved by the Government of Lower Franconia, Germany (Regierung von Unterfranken, RUF 55.2.2–2532–2-771–21). The researcher was blinded to the experimental conditions.

### 2.3 Experimental design

Forty-two animals were divided into different treatment groups receiving either DMSO or BTZ (0.2 mg·kg^−1^) and were evaluated at two different time points: day 11 (*n* = 6) and day 25 for behavioral testing (*n* = 6). In addition, six animals were included as a naïve control group. We also added a separate group for BTZ and NfL measurements, with animals analysed at 30 min (*n* = 3) and 2 h (*n* = 3) after BTZ injection. Animals were housed three per cage. Under deep 2% isoflurane anesthesia, when there was no paw withdrawal upon pinching, animals were injected with either BTZ at a dose of 0.2 mg·kg^−1^ in DMSO or an equivalent volume of DMSO 5% intraperitoneally (i.p.). The administration schedule of BTZ was established based on clinical treatment regimens on days 1, 4, 8, and 11, as well as previous reports (Duggett and Flatters, 2017; Yamamoto et al., 2015). According to previous studies, the peak effect of BTZ was expected on day 11, which would be resolved by day 25 after the first injection (Atalla et al., 2026). After sacrificing animals at two time points (day 12 and 26), blood, skin, sciatic nerves, and DRG from L3 to L5 were harvested and collected for further analysis.

### 2.4 Mechanical hypersensitivity

Briefly, mechanical sensitivity was assessed by 50% paw withdrawal threshold (PWT) on days 0, 1, 11, 18, and 25 using the von Frey filaments (Aesthesio® set, UGO BASILE) (Atalla et al., 2026; Sauer et al., 2017; Ben-Kraiem et al., 2021; Hecker et al., 2023). Animals were habituated to the mesh-like testing cages for 4 consecutive days. On each habituation day, animals were placed individually in the cages for 15 min and allowed to acclimatize until they were calm and showed minimal exploratory activity (Fig. 1A). A series of von Frey filaments were applied on the plantar surface of the hind paw for 1–3 s based on the up-down method (Chaplan et al., 1994) starting with the intermediate 4 g filament. Following the first change in response direction, responses were recorded until at least six responses had been obtained. Each left or right hind paw was tested three times with an interval of at least 10 min between consecutive measurements. No significant difference was observed between the PWTs of the left and right hind paws; therefore, the mean of the three measurements from each paw was calculated, and the resulting values from both hind paws were averaged to obtain a single PWT value for each animal. The withdrawal threshold of the hind limbs to a mechanical stimulus was determined by using the 50% paw withdrawal threshold (PWT) method.

**Fig. 1:**
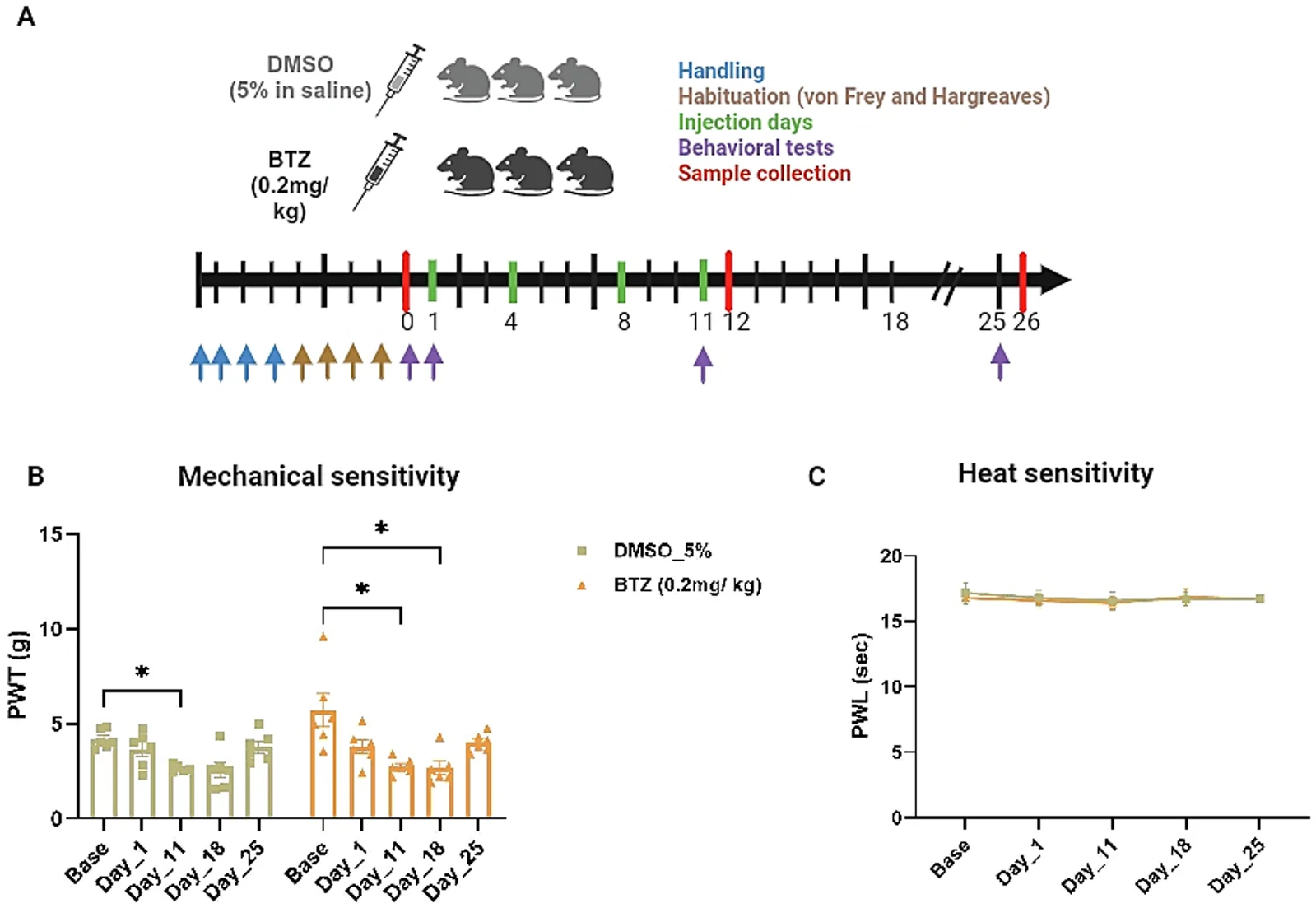
Effect of 0.2 mg·kg^−1^ BTZ or vehicle (DMSO) administration on mechanical and thermal hypersensitivity in male Wistar rats. A. The study workflow includes four intraperitoneal (i.p.) injections of 0.2 mg/kg BTZ or 5% DMSO in male Wistar rats, behavioral assessments performed at baseline and at the indicated post-treatment time points, and tissue collection for molecular analyses. B. Normalized 50% paw withdrawal threshold before (base) and after injections of 0.2 mg·kg^−1^ BTZ or vehicle (DMSO), on days 0, 1, 11, 18 and 25. *p < 0.01 DMSO at day 11 compared to baseline, *p < 0.04 BTZ at day 11 compared to baseline, *p < 0.01, BTZ at day 18 compared to baseline, data presented as the mean ± *SEM*, *n* = 6 per group. C. Paw withdrawal latency (PWL) in seconds to a radiant heat stimulus before (base) and after injections of 0.2 mg·kg^−1^ BTZ or vehicle (DMSO), on days 0, 11, 18 and 25. Data are presented as mean ± *SEM*, *n* = 6 per group.

### 2.5 Thermal hyperalgesia

Thermal sensitivity was assessed using the Hargreaves plantar test (IITC, Woodland Hills, CA, USA), originally described by Hargreaves et al. (Hargreaves et al., 1988) on days 0, 1, 11, 18, and 25. Animals were handled for 4 consecutive days before behavioral testing to familiarize them with the experimenter. They were subsequently habituated to the Hargreaves apparatus for 4 consecutive days by placing them individually in the glass cages and were allowed to acclimatize for 15 min to the testing environment (Fig. 1A). The hind paw withdrawal latency to the heat source was recorded. The active intensity of the light was set at 50% maximum. The test was repeated three times on each left or right hind paw, and the time difference between each trial was at least 10 min and cut-off latency set to 20 s to avoid any tissue damage. The mean of all six measurements from one animal (*n* = 6 per group) was calculated by adding the latencies for each left or right hind paw and averaging them (Chen et al., 2021; Atalla et al., 2026).

### 2.6 Serum neurofilament light protein measurements

Animals were euthanized by CO₂ inhalation at each designated sampling time point. Immediately thereafter, blood samples were collected by cardiac puncture, with a minimum of 1 mL of blood collected from each animal. After storing for 30 min at room temperature, the samples were subsequently centrifuged at 2655 RCF and 4 °C for 10 min, and the resulting serum was carefully collected and stored at −80 °C until analysis. In addition, serum was collected from animals at 2 h (after one BTZ or vehicle injection), 12 days (after four injections), and 25 days following the first injection. The next-generation Enzyme-Linked Immunosorbent Assay (ELISA), the ELLA™ device (Protein Simple, CA, USA), was used to measure NfL levels according to the manufacturer's instructions. Quantification range was 2.7–10, 290 pg/ml, *n* = 3–6 per group.

### 2.7 RNA reverse transcription qPCR (RT-qPCR)

Total RNA was isolated using the Qiagen miRNeasy Minikit (1,038,703, Qiagen, Hilden, Germany). Animals were sacrificed on days 12 and 25 after the beginning of the study, and left sciatic nerve samples from DMSO- and BTZ-treated rats (*n* = 6 per group) were collected. Tissue samples were homogenized in 700 μL QIAzol Lysis Reagent (Cat. No. 58107991, Qiagen, USA) using a homogenizer disperser (MICCRA D-9, Germany) for 1 min. After incubation for 5 min at room temperature, 140 μL chloroform was added, followed by incubation for 2–3 min. Samples were then centrifuged at 12,000 ×*g* for 15 min at 4 °C. The resulting aqueous phase was collected, mixed with 525 μL of 100% ethanol, and vortexed. A volume of 700 μL of the resulting mixture was transferred to an RNasy mini column and centrifuged by 8000 ×*g* at room temperature (RT) for 20 s. The column was subsequently processed according to the manufacturer's protocol, including the recommended washing and centrifugation steps. RNA was eluted by applying 33 μL RNase-free water directly onto the membrane and centrifuging at 8000 ×g for 1 min. RNA concentration and purity were assessed using a NanoDrop One spectrophotometer (Life Technologies GmbH, Darmstadt, Germany).

For cDNA synthesis, 250 ng of total RNA was reverse transcribed in a final volume of 100 μL with TaqMan Reverse Transcription reagents (Thermo Fisher Scientific, Waltham, MA, USA). The reaction mixture contained 10 μL 10× reaction buffer, 22 μL MgCl₂, 20 μL dNTP mix, 2 μL RNase inhibitor, 5 μL random hexamers, 6.25 μL MultiScribe reverse transcriptase, and nuclease-free water (NFW). Reverse transcription was performed under the following cycling conditions: annealing at 25 °C for 10 min, reverse transcription at 37 °C for 60 min, and enzyme inactivation at 90 °C for 5 min.

Target genes tagged with FAM-MGB reporter dye with following primers: IL-6 (Rn01410330_m1), IL-1ß (Rn00580432_m1), and the endogenous control GAPDH tagged with VIC-MGB reporter dye (Rn01462662_g1). Reactions were run in MicroAmp Fast 96-well reaction plates (4,346,907, Applied Biosystems, Darmstadt, Germany). Each reaction contained 3.5 μL of cDNA, 0.5 μL of primer, 5 μl of TaqMan® Fast Advanced Master Mix (2×) (4,444,558, Applied Biosystem, Darmstadt, Germany) and 1 μL of NFW, with reactions performed in duplicate. Real-time PCR was carried out using a StepOnePlus™ Real-Time PCR System (Applied Biosystems, Darmstadt, Germany) under the following settings: 2 min, 50 °C; 2 min, 95 °C; (3 s, 95 °C; 30 s, 60 °C) 40× and raw data were acquired with the StepOne software v2.3. Relative gene expression was analysed according to the ΔΔCt method, data are given as relative quantity (RQ) values (García-Fernández et al., 2023).

### 2.8 Liquid chromatography tandem mass spectrometry (LC-MS/MS)

We performed LC-MS/MS to measure levels of BTZ in the serum at 30 min and 2 h after the first injection, and four injections on days 12 and 25. In brief, BTZ and its metabolites, M1 and M2, as well as the internal standard BTZ-d8, were obtained from Biozol Diagnostica (Hamburg, Germany). Calibration samples were prepared in human serum as a surrogate matrix. Stock solutions of BTZ, M1, M2, and BTZ-d8 were prepared in methanol, and were then combined in a 100-fold concentrated calibration curve ranging from 52,632 ng/mL to 51 ng/mL (BTZ) or 23,616 ng/mL to 26 ng/Ml (M1/M2) in methanol (11 calibration levels). Before the preparation of rodent samples, the concentrated calibration curve was used to spike human serum at a ratio of 1:100 (990 μL serum +10 μL calibration stock solution). Rodent samples and calibration standards (50 μL) were precipitated with acetonitrile containing 0.1% (*v*/v) formic acid and BTZ-d8, vortexed, and then centrifuged. The supernatant was subsequently injected into the LC-MS/MS system. Samples were analysed on an X500R qTOF mass spectrometer (Sciex) coupled to an Agilent 1290 UHPLC. The separation was performed on a Waters XSelect CSH C18 column (2.1 × 50 mm, 3.5 μm particle size). A gradient elution of water (0.1% formic acid, phase A) and methanol (0.1% formic acid, phase B) was applied at a flow rate of 0.8 mL/min. The eluent was directed to the mass spectrometer between 1.6 and 2.8 min.

Compounds were detected and quantified by high-resolution multiple reaction monitoring (MRM), with precursor ions selected by the quadrupole and fragment ions analysed by TOF at a mass accuracy of <20 ppm. ESI source conditions were: curtain gas 25 psi, GS1 40 psi, GS2 50 psi, source temperature 500 °C, CAD gas 7 a.u., ionspray voltage 2000 V, positive ion mode. The monitored transitions were *m*/*z* 381.2 → 226.094 for BTZ, *m*/*z* 339.2 → 226.094 for M1/M2, and *m*/*z* 389.2 → 234.144 for the internal standard BTZ-d8. M1 and M2 shared the same MRM transition due to their identical *m*/*z* values and fragmentation patterns. BTZ-d8 served as the internal standard for all analytes. Although M2 was chromatographically separated, it was not quantified due to its low sensitivity.

### 2.9 Immunostaining

DRG (L3-L5) and right sciatic nerve samples were removed and embedded in Tissue Tek O.C.T. compound, and snap frozen in liquid nitrogen. Hind paw skin tissue was fixed for 1 h in 4% paraformaldehyde/ Phosphate-Buffered Saline (PFA/ PBS) at RT and dehydrated 30% using sucrose/ PBS at 4 °C overnight. On the following day, the skin samples were embedded in cold methylbutane and snap-frozen in liquid nitrogen. DRG sections were cut at 14 μm, nerve samples at 10 μm and skin at 40 μm thickness using a cryostat (Leica Microsystems, Wetzlar, Germany) at −22 °C. Tissue sections from DRG and nerve were post-fixed for 30 min in 4% PFA at RT and washed with PBS. DRG and skin samples were incubated for 1 h with blocking solution containing 10% donkey serum (DS) and 0.3% Triton X-100 in PBS. Slides were incubated with primary antibodies prepared in 1% DS including rabbit anti-PGP9.5 (1:500, Abcam, ab108986), mouse anti-NeuN (1:400, Abcam, AB104244), chicken anti-GFAP (1:1000, Novus Bio, NBP1–05198), rabbit anti-CGRP (1:500, BMA Biomedicals, T-4032), overnight at 4 °C. Sections were washed with PBS and the appropriate secondary antibody diluted in 1% DS and 0.3% Triton X-100 in PBS. Alexa Fluor® 647 AffiniPure-Donkey Anti-Rabbit IgG (1:1000, Jackson, 711–605-152) for PGP9.5 and CGRP, Cy3-donkey anti-Mouse IgG (1:1000, Jackson, 715–165-151) for NeuN, Alexa Fluor® 488 AffiniPure-donkey anti-chicken IgG (1:1000, Jackson, 703–545-155) for GFAP were added for 1 h at room temperature. After washing with PBS, cell nuclei were stained using 4′,6-diamidino-2-phenylindole (DAPI) and images were taken by a Leica Thunder Imager (Leica Microsystems, Wetzlar, Germany).

Right proximal sciatic nerves processed for macrophage detection. Sections were blocked for 30 min in 10% BSA prepared in Tris buffer (12 mM, pH = 7.4), then incubated overnight at 4 °C with the primary antibody mouse anti-rat ED1 (1:500, MAK0341, Serotec, Oxford, UK). On the following day, sections were first washed then incubated for 20 min in a solution containing 200 mL methanol and 3.5 mL of 30% hydrogen peroxide at RT to quench endogenous peroxidase activity. Sections were subsequently washed three times with Tris buffer. The biotinylated secondary antibody solution was prepared by mixing biotinylated goat anti-mouse IgG (BA-9200, Vector Laboratories, California, USA) with undiluted normal rat serum at a 1:1 ratio and incubating the mixture for 1 h at 37 °C. The resulting mixture was subsequently diluted 1:100 in 1% BSA/ Tris buffer and applied to the sections for 45 min at room temperature. Vector Laboratories ABC kit (PK-4000, Vector Laboratories, California, USA) solutions A and B were mixed at a 1:1 ratio and incubated for 30 min at room temperature. The resulting ABC solution was then diluted 1:50 in 1% BSA/Tris buffer and applied to the sections for 30 min at room temperature. Sections were subsequently washed three times with Tris buffer. Immunoreactivity was visualized using incubation with DAB buffer (SK-4100, Vector Laboratories, California, USA) for 10 min at RT in the dark. Sections were then washed twice with distilled water and counterstained with Mayer's hematoxylin for 45 s. Slides were rinsed in running water for 10 min, dehydrated through a graded ethanol series (70%, followed by 100% ethanol ×3), and cleared in xylene for 5 min.

Macrophage abundance was quantified as the percentage of macrophages per area (cells/ mm^2^), see 2.11).

### 2.10 Semithin sections

To investigate whether BTZ administration induces myelin disruption, right distal sciatic nerve samples were incubated in 4% paraformaldehyde plus 2.5% glutaraldehyde solution for 24 h. Samples were postfixed in 2% osmium tetroxide (OsO4) prepared in 0.1 M sodium cacodylate (NaCaCo) buffer for 90 min at RT. Following postfixation, samples were washed with 0.1 M NaCaCo buffer and incubated overnight with it at 4 °C. On the following day, samples were dehydrated through a graded acetone series, with each concentration applied for 15 min, from 30% to 100%, with the 100% acetone step repeated twice. Samples were then infiltrated with epoxy resin (ERL) using a stepwise acetone/ ERL series. Samples were first incubated twice in acetone and once in ERL for 1 h, followed by incubation in acetone/ ERL at a ratio of 1:2, and finally in pure ERL overnight at 4 °C. In the next day, the samples were subsequently polymerized at 70 °C 24 h.

Following polymerization, resin-embedded nerve samples were sectioned at a thickness of 0.5 μm using a Leica EM UC7 ultramicrotome (Leica Microsystems, Wetzlar, Germany). Sections were stained for 90 s with a methylene blue-Azur II staining solution containing 1% methylene blue, 1% Azur II, and 40% sucrose in distilled water, with the solution adjusted to pH 7.4. Following staining, sections were dried on a heating plate at 80 °C and visualized using a Leica THUNDER Imager with a 20× objective.

### 2.11 Quantification of immunofluorescence and immunohistochemical staining

All sections were examined using a Leica THUNDER Imager 2 (Leica Microsystems, Wetzlar, Germany). Image acquisition parameters were kept constant throughout the analysis, including the objective, excitation light intensity using a stable LED light source, exposure time, bit depth, camera binning, and gain settings. The experimenter was blinded to the treatment groups during image acquisition and analysis. Images were acquired using Leica Application Suite X software (Leica Microsystems, Wetzlar, Germany).

For intraepidermal nerve fiber density (IENFD) analysis, three sections per slide were imaged. IENFD was determined by counting PGP9.5-immunoreactive nerve fibers crossing the dermal-epidermal junction and expressed as the number of fibers per millimeter of footpad skin section length. Images were acquired using a × 200 objective, as previously described (Mangus et al., 2020).

Positive cells in the DRG were quantified using a semi-automated approach with the Trainable Weka Segmentation plugin in ImageJ/Fiji (Arganda-Carreras et al., 2017). DRG sensory neurons were identified by NeuN immunoreactivity as a pan-neuronal marker for the detection of both large- and small-diameter neurons. CGRP immunoreactivity was used to identify peptidergic sensory neurons. Sections were imaged at ×200 magnification. For each staining, 2–3 sections per animal were analysed. A minimum of 80 NeuN neurons per section was required for inclusion; sections containing fewer than 80 neurons were replaced with another section from the same animal.

For each section, cells were counted measured using ImageJ/Fiji. Cell counts were normalized to the total number of neurons.

For GFAP analysis, SGCs were distinguished from neuronal somata and other perineuronal cells based on their morphology, anatomical position, orientation, and nuclear characteristics (Pannese, 2013). Sections were imaged at ×100 magnification.

Neuronal profiles surrounded by GFAP-positive SGCs of their circumference were considered positive. The number of GFAP-positive neuronal profiles was quantified and expressed as a percentage of the total number of neurons analysed.

Bright field images were acquired using Leica Application Suite X software (Leica Microsystems, Wetzlar, Germany) at ×200 magnification. ED1-positive macrophages were identified and counted in the analysed sections. Cell counts were normalized to the analysed tissue area.

### 2.12 Statistical analysis

All statistics were performed using Graph Pad Prism software 9.4.1 (San Diego, CA, USA). Behavioral results were analysed by two-way RM ANOVA followed by Green-house Geisser correction. GFAP, CGRP, NeuN and gene expression were analysed using one-way ANOVA. Macrophage abundance was assessed by two-way ANOVA. Kruskal-Wallis nonparametric test was used for IENFD, serum NfL and BTZ level analysis. *P*-value <0.05 was considered statistically significant.

## 3 Results

### 3.1 Weight gain, mechanical hypersensitivity and thermal hyperalgesia after BTZ and DMSO administration

The experimental design is shown in Fig. 1A. Rats treated with BTZ or DMSO showed weight gain that increased steadily over time (Fig. S1). The mechanical withdrawal thresholds to plantar stimulation with von Frey hairs dropped slowly at one day after the first injection and significantly at day 11 in the DMSO treated group versus baseline (*p* = 0.01), and in BTZ treated animals at both day 11 and day 18 (*p* = 0.04 and 0.01) compared to baseline, with resolution by day 25 (Fig. 1B) Sensitivity to heat (Fig. 1C), remained unchanged over time.

### 3.2 Serum NfL and BTZ levels

NfL was measured in serum at 2 h, 12 days, and 25 days following BTZ injection. There was no increase compared to naïve animals at any time point in either group (Fig. 2A).

**Fig. 2:**
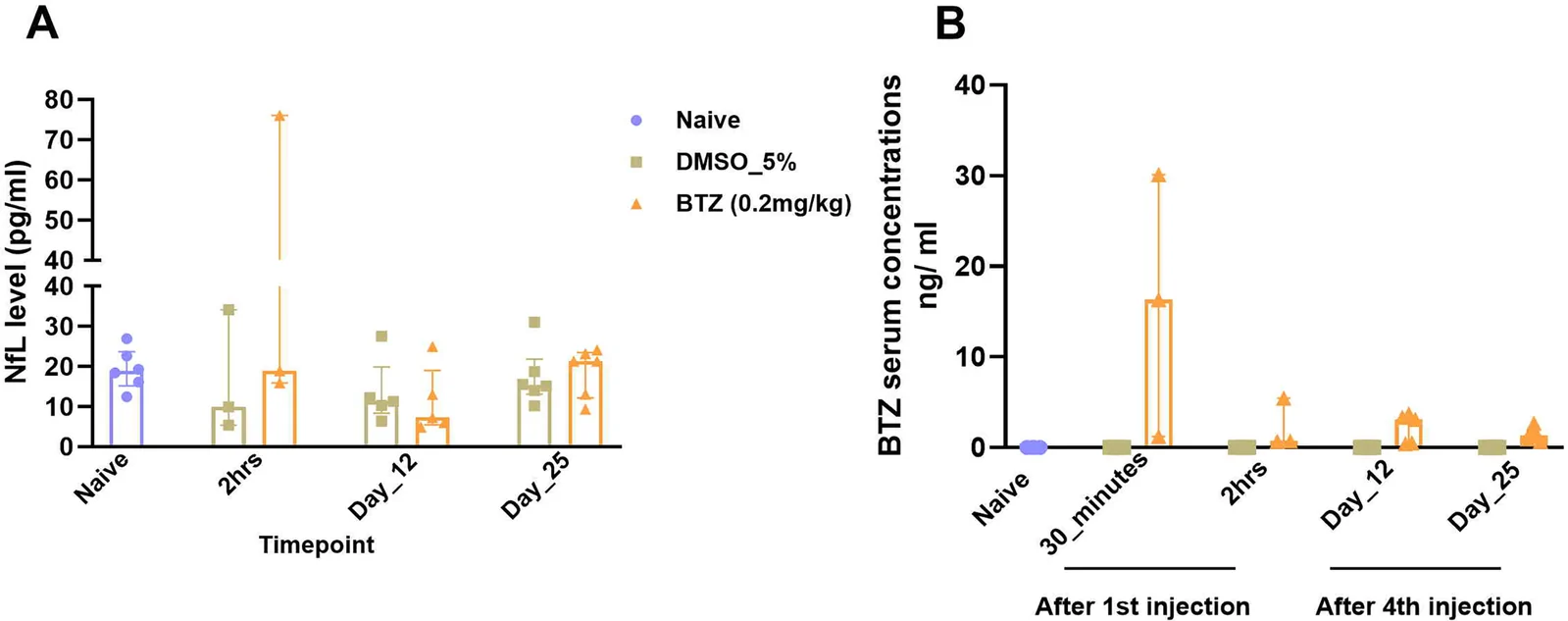
A. NfL levels in rat serum from naïve animals and at 2 h, 12 days, and 25 days following injection of 0.2 mg·kg^−1^ BTZ or vehicle (DMSO). Data presented as the median and percentiles, *n* = 3 at 30 min and 2 h after the injection, *n* = 6 in naïve, day_12 and day_25 groups. B. Serum concentration of BTZ after a single (30 min and 2 h) and four (day_12 and day_25) injections of 0.2 mg·kg − 1 BTZ or DMSO. Data presented as the median and percentiles, *n* = 3–6 per group.

We performed LC-MS/MS to measure BTZ levels in the serum at 30 min and 2 h after the first injection, and on day 12 (1 day after last injection) and day 25 (14 days after last injection). BTZ was present in serum as early as 30 min after the first i.p injection and could be found until day 25 (Fig. 2B).

### 3.3 Skin intraepidermal nerve fiber density

When evaluating IENFD in footpads as an early sign of neuropathy (Sommer and Lauria, 2007) following BTZ/ DMSO administration, using PGP9.5 immunofluorescence as shown in Fig.3A and B in green, we found a trend toward a decrease in IENFD from baseline to day 12 and 25 days in both groups (n.s.) but no difference between DMSO and BTZ treated animals (Fig. 3C).

**Fig. 3:**
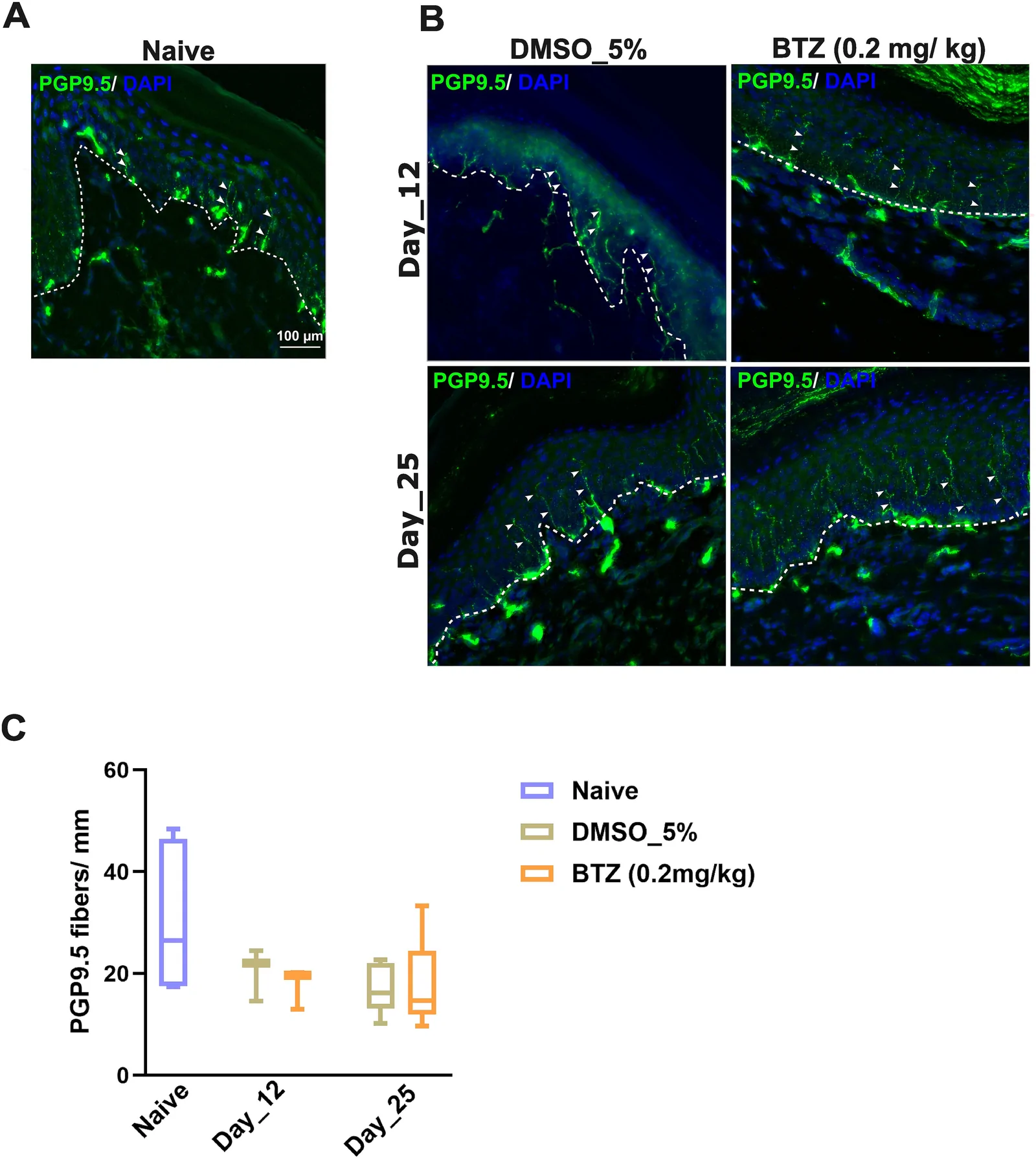
Representative images of intraepidermal nerve fiber density (IENFD) evaluation with PGP9.5 immunofluorescence staining on footpad skin from naïve rats and after four injections of 0.2 mg·kg^−1^ BTZ or vehicle (DMSO), on days 12 and 25. A and B immunostaining of PGP9.5 (green) and DAPI (blue) on foot pad skin from the naïve, BTZ or DMSO treated animals, respectively. Examples of nerve fibers are indicated by arrowheads. C. Quantification of IENFD. Data presented as the median and percentiles. Scale bar = 100 μm, *n* = 3 on day 12 and *n* = 5 on day 25 and for naïve rats. (For interpretation of the references to colour in this figure legend, the reader is referred to the web version of this article.)

### 3.4 Sciatic nerve analysis

We checked sciatic nerve for macrophage infiltration using ED1 as a macrophage marker. Quantitative analysis revealed that both groups' nerves contained similar numbers of macrophages at both 12 days and 25 days after injection, (Fig. 4A and B). Naïve nerve samples were examined qualitatively to confirm the presence of ED1-positive macrophages.

**Fig. 4:**
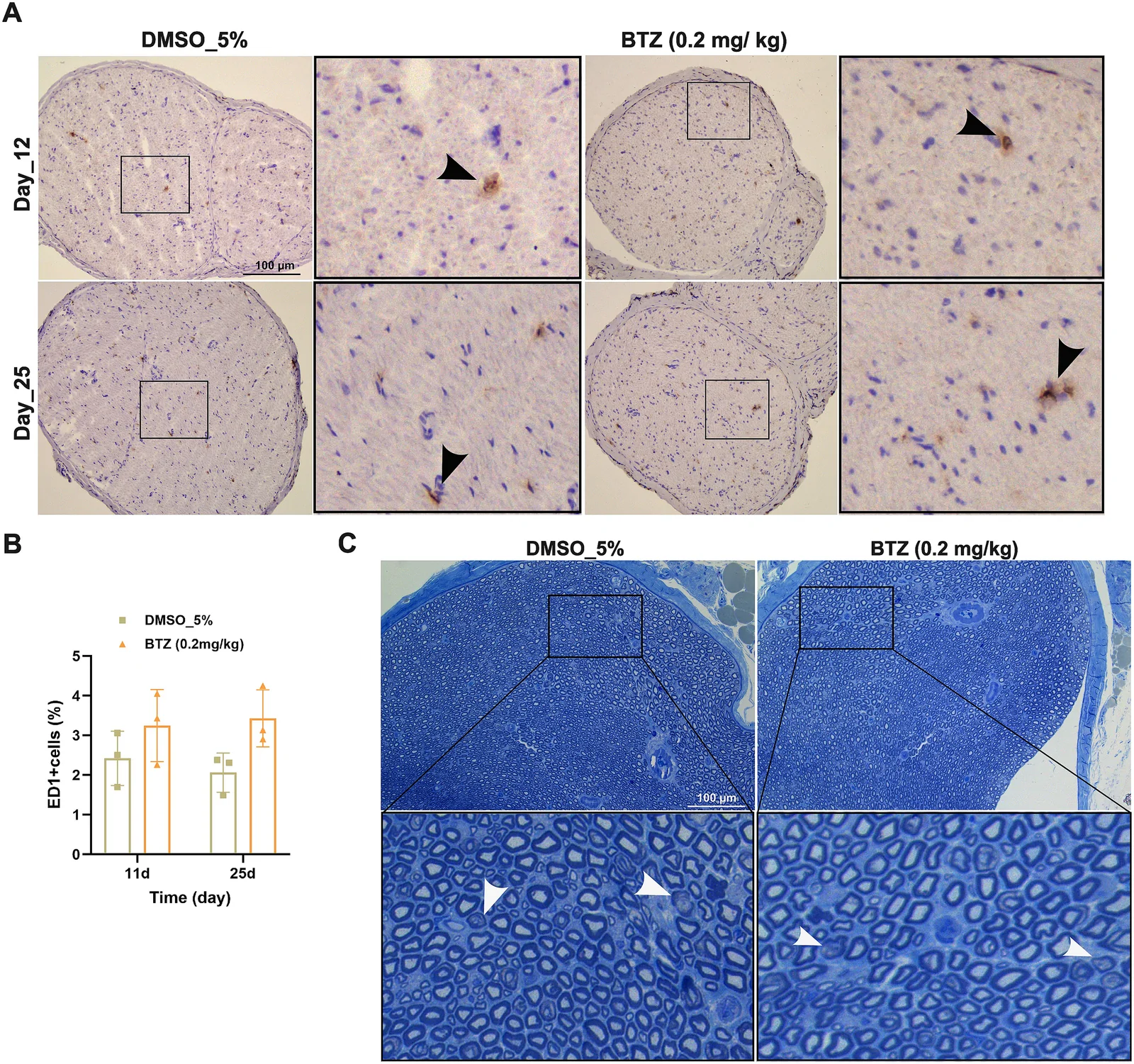
Light photomicrograph, sciatic nerves from Wistar rats. A. Macrophage infiltration is shown using immunostaining against ED1 (arrows) on days 12 and 25 after four injections of 0.2 mg·kg^−1^ BTZ or vehicle (DMSO), scale bar = 100 μm, *n* = 3. B. Quantification of macrophages in an area. C. Semithin sections of sciatic nerve after four injections of 0.2 mg·kg-1 BTZ or vehicle (DMSO), arrowheads show disrupted myelin, Scale bar = 100 μm, *n* = 3.

Semithin sections of sciatic nerve obtained at 25 days after injection did not show signs of nerve degeneration except for occasional myelin disruption in both groups (Fig. 4C).

### 3.5 Satellite glial cell activation

Immunofluorescence staining was performed on L4-L5 DRG for GFAP (to stain activated satellite cells in red) along with NeuN (neuronal marker in cyan) (Schulte et al., 2023). The resulting images were analysed quantitatively to assess the percentage of neurons surrounded by GFAP-positive cells to all neurons. GFAP immunofluorescence increased moderately at time point day 12 and 25 in the DMSO group compared to naive rats (*p* < 0.02; 0.01), and in BIPN animals at day 12 and 25 (*p* < 0.0001). A comparison between DMSO and BTZ treated animals on day 12 and day 25 timepoints revealed a notably higher percentage of neurons surrounded by GFAP under BTZ treatment (*p* < 0.006), this difference persisted and became even more pronounced by day 25 (*p* < 0.0001) (Fig. 5A and B).

**Fig. 5:**
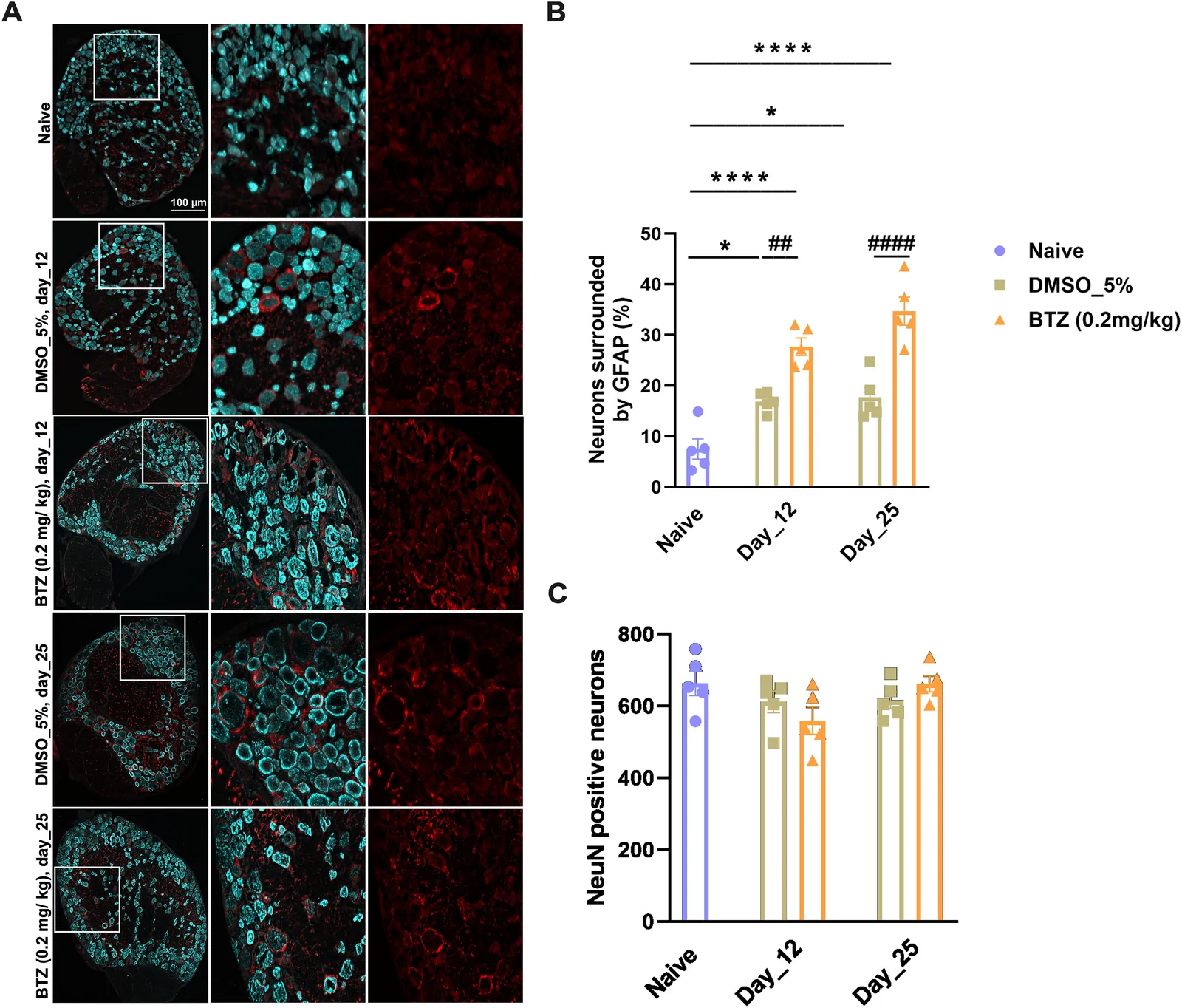
Representative tile-scan images of DRG sections from naïve Wistar rats or after four injections of 0.2 mg·kg^−1^ BTZ or vehicle (DMSO) on days 12 and 25. A. Single or double immunostaining of NeuN (turquoise) and GFAP (red). B. Quantification of the percentage of the neurons surrounded by GFAP positive satellite glia cells, *p < 0.02 DMSO, day 12 compared to naïve, ****p < 0.0001 BTZ, day 12 compared to naïve, *p < 0.01 DMSO, day 25 compared to naïve, ****p < 0.0001 BTZ, day 25 compared to naïve. ##*p* < 0.006 BTZ, day 12 compared to DMSO at day 12, ####*p* < 0.0001 BTZ, day 25 compared to DMSO at day 25. Data presented as the mean ± *SEM*, Scale bar = 100 μm, *n* = 5. C. Quantification of NeuN positive cells in neuron enriched area. Data presented as the mean ± *SEM*, Scale bar = 100 μm, *n* = 5. (For interpretation of the references to colour in this figure legend, the reader is referred to the web version of this article.)

### 3.6 Neuronal loss

To assess neuronal integrity of DRG neurons we counted NeuN positive cells. The number of NeuN positive cells in the whole DRG area or NeuN positive cell density did not differ between animals treated with BTZ and those treated with DMSO on days 12 or 25 or compared to naive animals (Fig. 5C).

### 3.7 Calcitonin gene related peptide analysis in DRG

Subsequently, we analysed DRG for peptidergic (CGRP positive) neurons (Fig. 6). BTZ treated rats had a higher number of CGRP-immunoreactive neurons at day 12 compared to the naive control animals (*p < 0.02). The numbers of CGRP positive neurons in the DMSO treated animals were not different to the naive group (Fig. 6A and B).

**Fig. 6:**
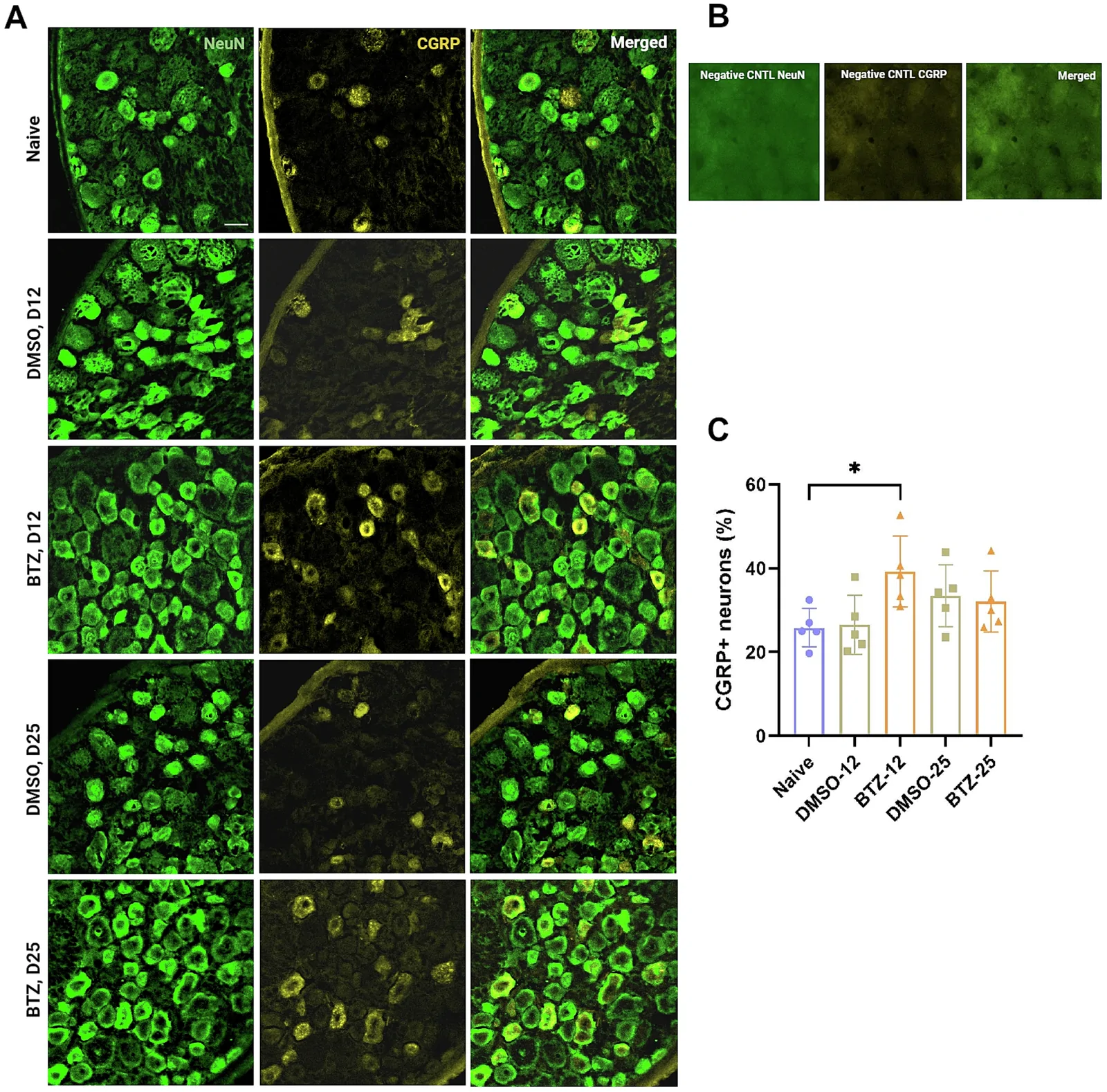
Representative immunofluorescent staining in DRG sections from Wistar rats after four injections of 0.2 mg·kg^−1^ BTZ or vehicle (DMSO) on days 12 and 25. A. Representative images of double immunostaining for CGRP (yellow), a marker of peptidergic neurons, and NeuN (green), a pan-neuronal marker. B. Representative images of the corresponding negative controls for CGRP and NeuN staining. C. Quantification of CGRP positive neurons per number of neurons (^⁎^*p* < 0.02 BTZ 0.2 mg·kg^−1^, day_12 compared to naive,). Data presented the mean ± *SEM*, Scale bar = 100 μm, *n* = 5 per group. (For interpretation of the references to colour in this figure legend, the reader is referred to the web version of this article.)

### 3.8 Cytokine expression

In the next step, IL-6 and IL-1ß gene expression was assessed in the sciatic nerve as shown in Fig. 7. RQ values indicated a trend toward increased IL-6 expression in BTZ treated animal at day 25 (*p* = 0.09) in comparison to the naïve animals, Fig. 7A. However, this difference did not reach statistical significance. No significant changes in IL-1β expression were observed at any time point, Fig. 7B.

**Fig. 7:**
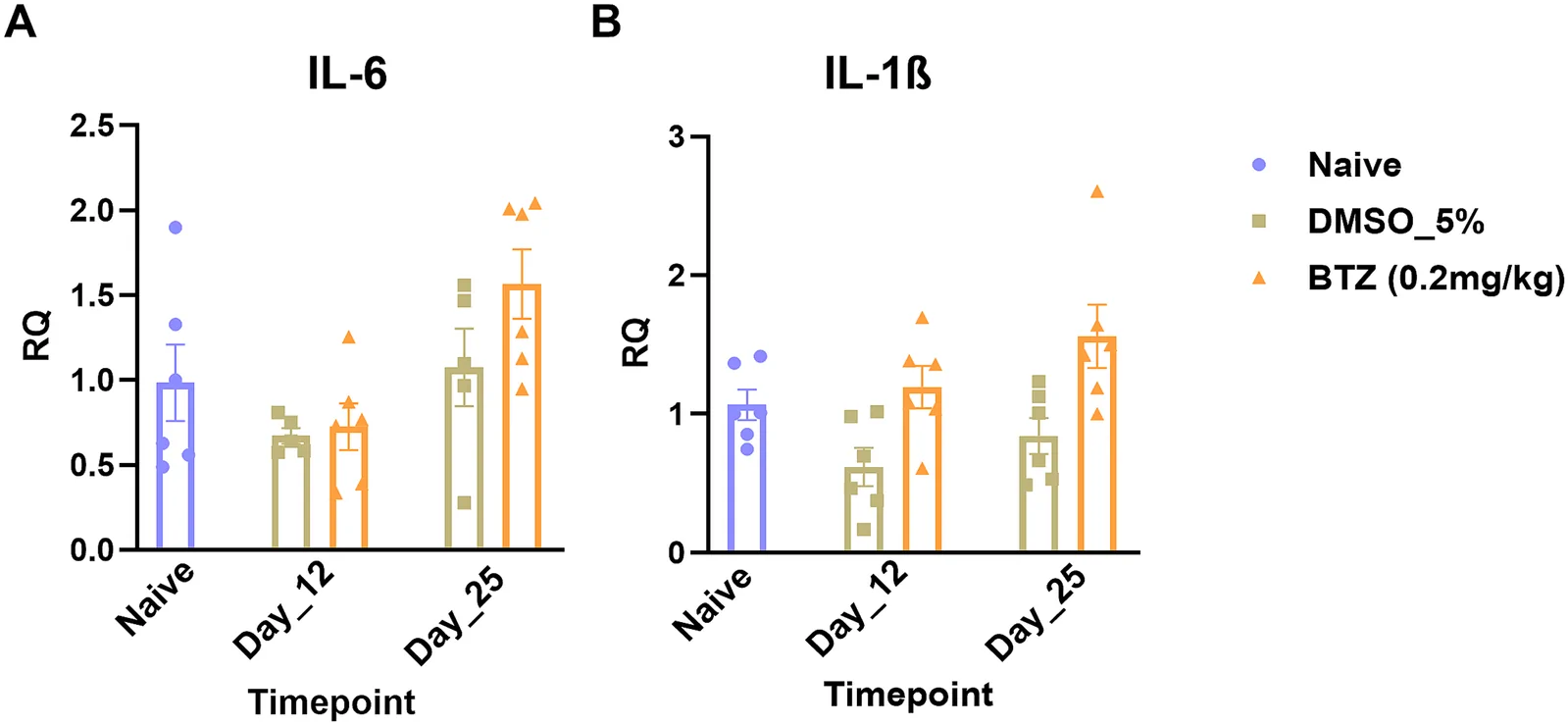
Relative IL-6 (A) and IL-1ß (B) gene expression in sciatic nerve from Wistar rats after four injections of 0.2 mg·kg^−1^ BTZ or vehicle (DMSO) on days 12 and 25. Data presented as the mean ± *SEM*, *n* = 6 per group.

## 4 Discussion

The aim of this study was to investigate mechanisms of BIPN development and resolution in a rat model, using a clinically relevant BTZ dosing regimen as previously described (Yamamoto et al., 2015). BTZ was present in the bloodstream within 30 min post-injection and remained at low levels up to day 25, i.e. 14 days after the last injection.

Rats developed transient allodynia after DMSO, whereas BTZ induced a mild, reversible pattern of mechanical hypersensitivity consistent with functional sensitization that peaked around day 12 and subsided by day 25. We did not detect structural damage in footpad skin or sciatic nerves. At day 12, only BTZ rats displayed an increase in CGRP-immunoreactive neurons in the DRG, which normalized by day 25 in line with behavioral recovery. In contrast, SGC activation was evident in both BTZ- and DMSO-treated animals and remained elevated through day 25, suggesting persistent glial activation despite behavioral resolution.

### 4.1 Both DMSO and BTZ induce mild mechanical hypersensitivity

Consistent with prior studies, no differences were observed in thermal nociceptive thresholds in DMSO or BTZ-treated animals, indicating that heat sensitivity remains largely unaffected (Meregalli et al., 2010). A transient but significant reduction in mechanical withdrawal threshold was observed in animals treated with 5% DMSO, indicating a slight increase in mechanical sensitivity over time. This observation is consistent with previous reports indicating that DMSO at concentrations exceeding 3.6% may produce mechanical allodynia or enhance nociceptive sensitivity (Cavaletti et al., 2000; Authier et al., 2002).

In contrast, BTZ-treated animals exhibited a mild but more persistent mechanical allodynia, which peaked between days 11 and 18 and had resolved by day 25, confirming previous findings (Atalla et al., 2026). This sustained hypersensitivity is in line with the subclinical neuropathy that can be observed in most BTZ-treated patients (Yamamoto et al., 2015).

Other contributing factors to the observed drop in threshold in both groups may include mild testing-related stress, repeated handling, or physiological fluctuations in pain perception over the experimental period (Thomsen et al., 2025). Furthermore, all animals were exposed to isoflurane anesthesia prior to injection, which may have had an additive or sensitizing effect on nociceptive pathways. Isoflurane exposure has been shown to predispose animals to chronic pain phenotypes including mechanical allodynia (Li et al., 2019). While anesthesia and handling cannot be avoided when conducting these experiments, we concluded to not use DMSO as a vehicle in future behavioral experiment.

### 4.2 Skin innervation and sciatic nerve integrity are not affected in this BIPN model

Neither DMSO nor the cumulative dose of BTZ, administered at 0.8 mg/kg using a clinically relevant schedule, induced measurable epidermal denervation. The absence of IENFD loss is consistent with previous reports using the clinical formulation of bortezomib (Velcade®), where behavioral hypersensitivity was observed without accompanying structural loss of intraepidermal fibers (Cebulla et al., 2023). In contrast, some IENFD reduction at the peak of mechanical sensitivity was found in a previous study using the same treatment regime (Atalla et al., 2026), and a 25% loss of IENFD after administration of 1.0 mg/kg cumulative dose of BTZ on 5 consecutive days has been reported at the time of peak pain severity (Zheng et al., 2012). Also, rats receiving a cumulative dose of 4.8 mg/kg of BTZ i.v. had a significant decrease in IENFD (Meregalli et al., 2018). Thus, in addition to a probable dose dependency, there is considerable variation in severity of the model even when using the same treatment regime. These findings further support that the present low-dose BTZ paradigm reflects a predominantly functional and reversible neuropathy rather than a model of overt neurodegeneration.

Sciatic nerve assessment showed signs of mild myelin disruption, as shown in Fig. 4. Notably, similar structural alterations were observed in DMSO-treated animals, including axonal swelling accompanied by signs of myelin wrinkling. These findings support earlier studies indicating that administration of DMSO can induce peripheral nerve dysfunction and axonal damage (Cavaletti et al., 2000). DMSO at concentrations of 0.5% or higher can disrupt neuronal morphology in culture and impair the viability of neuronal (Hanslick et al., 2009) and non-neuronal cells (Yuan et al., 2014) and has been shown to reduce nerve conduction velocity in the rat sciatic nerve in a dose-dependent manner due to myelin breakdown (Cavaletti et al., 2000).

Macrophage analysis in both the BTZ and DMSO groups revealed no significant differences, with similar ED1-positive cell distribution observed at days 12 and 25 post-injection. Importantly, resident macrophages are normally present throughout the sciatic nerve in naïve animals (Mueller et al., 2001). In contrast, an increase of macrophages in sciatic nerves after BTZ injection has been shown in a previous study using a 4.8 mg/kg cumulative dose (Meregalli et al., 2018). Therefore, our findings suggest that the mild macrophage activation observed at the lower BTZ dose used here may reflect a dose-dependent response and also emphasize the need to account for DMSO-induced inflammatory effects when interpreting myelin structure and macrophage involvement in neuropathy models.

### 4.3 Serum NfL levels indicate a lack of BTZ-related axonal damage

To complement the histological assessment, serum NfL levels were measured at multiple time points. NfL is a well-established biomarker of axonal damage and neurodegeneration in both peripheral and central nervous system disorders (Khalil et al., 2018), and has been shown to indicate neurotoxicity in MM patients undergoing BTZ-based therapy (Cebulla et al., 2023). In the present study, serum NfL concentrations remained stable across all time points following BTZ injection, indicating that the administered dose did not produce detectable axonal injury. This finding is in accordance with recent evidence that rats administered a cumulative BTZ dose of 1 mg/kg did not display any increase in blood NfL, in contrast to other neurotoxic chemotherapies, such as paclitaxel and vincristine, which markedly elevate circulating NfL (Balayssac et al., 2023). In the therapeutic context, it is noteworthy that blood NfL concentrations have been observed to be higher in patients with MM receiving continuous BTZ treatment than in those tested after medication is finished (Cebulla et al., 2023). This suggests that peak neurotoxicity markers may coincide with the cumulative dose of BTZ in the blood.

### 4.4 Absence of neuronal loss but increase in CGRP-immunoreactive neurons and activation of glial cells under the experimental conditions

Neuron density as assessed by NeuN quantification upon BTZ at a cumulative dose of 0.8 mg/kg and DMSO 5% treatment revealed no significant change in total neuronal counts, suggesting preservation of neuronal integrity under these conditions. These findings are consistent with previous reports showing that BTZ, when applied at clinically relevant doses, does not induce pronounced axonopathy in cultured DRG neurons (Zhou et al., 2023). Instead, it modulates pain-associated ion channels, such as TRPA1 and TRPV1, in sensory neurons from animals (Quartu et al., 2014). In addition, DMSO at a dose of 0.5% or higher (Zhang et al., 2017) can also damage neuronal morphology and impair their viability. In the present study, we found neither loss of sensory neuron cell bodies in the DRG nor a decrease in IENFD in the skin. Together, these findings strongly support the conclusion that functional impairments to sensory fibers, rather than overt denervation, may underlie BTZ-induced pain at lower, clinically relevant doses.

Given the dissociation between behavioral hypersensitivity and structural nerve degeneration, we next sought to examine other cellular components and neuroimmune mechanisms potentially driving BIPN. Specifically, we focused on neurochemical changes in sensory neurons, such as CGRP expression, and the activation of non-neuronal cells, including macrophages and SGC, which have emerged as critical modulators of peripheral nerve inflammation and sensitization. CGRP-positive neurons were significantly increased in BTZ-treated animals at day 12 compared to naïve animals, suggesting a shift in nociceptive signaling and increased peripheral sensitization. CGRP is a key mediator of neurogenic inflammation (Liu et al., 2016) and has been implicated in various models of chemotherapy-induced peripheral neuropathy (Akhilesh et al., 2024). The notion that CGRP mediates the onset and maintenance of early-phase mechanical hypersensitivity (Malon and Cao, 2016) but is not necessary for the late or resolved phase is supported by the absence of sustained CGRP elevation at day 25 in our study, which aligns with the time course of mechanical sensitivity.

GFAP immunoreactivity in the DRG was observed in both BTZ- and DMSO-treated animals compared to naïve controls, indicating SGC activation with a more pronounced increase in following BTZ treatment (Robinson et al., 2014; Yamamoto and Egashira, 2021). According to earlier research, GFAP overexpression in the spinal cord specifically appears in the early stages of BIPN and progressively decreases over time, reaching baseline levels by day 69 (Robinson et al., 2014). Rather than inducing neuronal damage, BTZ leads to SGC activation without inducing the release of inflammatory cytokines (Zhou et al., 2023). Interestingly, 5% DMSO treatment also increased GFAP immunoreactivity, which may be related to its ability to induce oxidative stress and mitochondrial dysfunction in glial cells. At concentrations exceeding 3%, DMSO has been shown to impair glial viability as well as mitochondrial membrane potential (Yuan et al., 2014). Additionally, it has been shown that DMSO treatment reduces the expression of retinoic acid, a neuroprotective metabolite that maintains glial cell homeostasis (Rabow et al., 2021), which may make SGCs more vulnerable to oxidative damage and inflammatory reactions.

The increase in GFAP expression in the early phase might be related to pain and CGRP neuronal hyperexcitability (De Logu et al., 2022). GFAP expression rose with time until day 25 despite the resolution of neurogenic inflammation and stable NeuN-positive neuron counts, which suggests persistent glial activation that may contribute to long-term modulation of sensory pathways in the peripheral nervous system.

### 4.5 No increase in cytokine expression in sciatic nerves after BTZ treatment

In our study, 0.8 mg/kg BTZ did not significantly increase the selected cytokine transcripts IL-1β or IL-6 gene expression in sciatic nerve. Mice treated with 1 mg/kg BTZ twice weekly for six weeks showed increased IL-6 levels in the DRG by the second week of treatment, whereas mechanical allodynia appeared earlier, on day four. This difference in timing suggests that IL-6 elevation is not required for the initial development of neuronal dysfunction but may play a role in maintaining the ongoing pain (Alé et al., 2014). BTZ at a cumulative dose of 2 mg/kg leads to IL-1ß increase as shown in the spinal dorsal horn as early as day four with the onset of mechanical allodynia (Li et al., 2016). Our analysis was limited to IL-1β and IL-6 gene expression in the sciatic nerve and selected time points. Therefore, the present data do not exclude the involvement of other inflammatory mediators, tissues, or signaling pathways in BTZ-induced neuropathy. Rather, these cytokine measurements provide an exploratory assessment of inflammatory gene expression under the experimental conditions used here.

### 4.6 Time-dependent serum concentrations of BTZ after i.p. injection

Our results demonstrated that BTZ was detectable in the bloodstream as early as 30 min after a single i.p. injection of 0.2 mg/kg, rising to approximately 20–30 ng/ml. Levels decreased at 2 h post-injection, but BTZ remained measurable until day 25, even 14 days after the last injection. Previously, only the very early course of BTZ blood levels in rodents has been described, as in a publication with similar findings at the 2 h timepoint as in the present study (Huehnchen et al., 2020).

Some discrepancies between studies impair direct comparisons. Variations result from differences in animal species, routes of administration (i.v. versus i.p. or s.c.), dosing schedules, cumulative doses, and drug formulations used (Höke, 2012). Thus, contradictory findings highlight the need for further species-dependent model standardization and optimization to ensure consistent results across studies (Cavaletti et al., 2000). In order to translate typical neurophysiological, behavioral, and clinical aspects associated with patients to animals, it has been suggested to use higher doses of BTZ and multiple administration times (Quartu et al., 2014; Meregalli et al., 2018). Differences in BTZ neurotoxicity between studies may also stem from variability in plasma concentrations. In patients, subcutaneous administration is associated with a lower incidence of peripheral neuropathy compared with intravenous delivery, consistent with reduced systemic exposure (Yamamoto and Egashira, 2021; Moreau et al., 2011). This variability underscores the importance of measuring and including serum BTZ levels in every BIPN model to accurately interpret neurotoxic effects and improve comparability across studies.

Using a clinically appropriate low-dose regimen, this study provides new insight into the mechanisms behind the onset and spontaneous remission of BIPN. Functional and cellular alterations, including transient upregulation of CGRP sensory neurons and persistent activation of SGCs emerge as key features of this model, however, the absence of detectable structural axonal loss as evidenced by stable IENFD, NeuN, and serum NfL levels indicates that BTZ neuropathy may be reversible if we can target maladaptive signaling early, also highlighting the need to focus not only on structural endpoints (cell loss) but also on neuron–glia communication.

Persistent SGC activation, even after the resolution of mechanical hypersensitivity, suggests that glial responses extend beyond the disappearance of behavioral symptoms and may modulate sensory pathways in the long term. Future studies should investigate whether this sustained SGC activation contributes either to maintaining resolution or, conversely, predisposes to relapse of neuropathic symptoms and altered sensory.

It should also be noted that DMSO commonly used as a vehicle or control in preclinical pain studies, may represent a confounding factor. In the present study, we showed that DMSO itself can influence behavioral outcomes, macrophage infiltration in the sciatic nerve and SGC activation in DRG. These findings highlight the importance of recognizing the potential effects of DMSO when interpreting experimental outcomes involving this solvent.

A limitation of our study is that we only used male rats. We started the experiment with male rats, because multiple myeloma and thus BIPN are more frequent in male than in female patients (Bird et al., 2021). We had planned to add a cohort of female rats in the second step, but given the subtle findings in this study and the DMSO effects, adhering to the 3R principles, we decided not to go ahead with this project. In addition, the relatively small sample size may have limited the sensitivity to detect subtle biological effects; therefore, negative findings should be interpreted with appropriate caution.

Overall, by establishing a low-dose, reversible rat model of BIPN that separates behavioral hypersensitivity from structural nerve loss, this work contributes to the field and offers a practical platform for researching spontaneous resolution processes and non-degenerative mechanisms of neuropathic pain.

## CRediT authorship contribution statement

**Mona Hashemian:** Writing – original draft, Methodology, Investigation, Formal analysis, Data curation. **Elisabete Bóf Ramos:** Writing – review & editing, Investigation. **Mariam Sobhy Atalla:** Writing – review & editing, Methodology, Investigation. **Nadine Cebulla:** Writing – review & editing, Investigation. **Jonas Huhn:** Writing – review & editing, Methodology, Investigation, Data curation. **Oliver Scherf-Clavel:** Writing – review & editing, Supervision, Methodology. **Grit Hein:** Writing – review & editing, Supervision. **K. Martin Kortüm:** Writing – review & editing. **Heike Rittner:** Writing – review & editing, Supervision, Resources, Conceptualization. **Claudia Sommer:** Conceptualization, Data curation, Funding acquisition, Methodology, Project administration, Resources, Supervision, Writing – original draft, Writing – review & editing.

## Declaration of competing interest

The authors declare that they have no known competing financial interests or personal relationships that could have appeared to influence the work reported in this paper.

## Data Availability

Data will be made available on request.
