# Supplementary material for: A rat model of mild, reversible bortezomib-induced peripheral neuropathy: behavioral resolution despite persistent glial activation
Source: Neurobiol Pain. 2026 Oct 27;20:100240. doi: 10.1016/j.ynpai.2026.100240 (PMC13756175; doi:10.1016/j.ynpai.2026.100240)
Supplement: Supplementary file 1 [file mmc1.docx]

**Supplementary figure**

**Figure S1**

Figure S1. Effect of 0.2 mg·kg^−1^ BTZ or vehicle (DMSO) administration on weight gain before (base) and after injections of 0.2 mg·kg^−1^ BTZ or vehicle (DMSO), on days 0, 11, 25. Data are presented as mean ± SEM, n = 6 per group.
